# Reinforcing Bus Living Space with Recycled Carbon Fibers from Expired Prepreg in the Aircraft Industry

**DOI:** 10.3390/ma17235958

**Published:** 2024-12-05

**Authors:** Miguel Angel Martínez, Daniel Lavayen-Farfán, Juana Abenojar, María Jesús López-Boada, Daniel García-Pozuelo

**Affiliations:** 1 Materials Science and Engineering Department, Universidad Carlos III de Madrid, 28911 Leganés, Spain; 2 Mechanical Engineering Department, Universidad Carlos III de Madrid, 28911 Leganés, Spain; dlavayen@pucp.edu.pe (D.L.-F.); mjboada@ing.uc3m.es (M.J.L.-B.); dgramos@ing.uc3m.es (D.G.-P.); 3 Applied Mechanics, Machines and Mechanisms Group, Pontificia Universidad Católica, Lima 15088, Peru; 4 Mechanical Engineering Department, Universidad Pontificia Comillas, 28015 Madrid, Spain

**Keywords:** metal structure enhancement, recycled CFRP prepreg, bending properties, bus living space, vehicle safety, passenger vehicles

## Abstract

Due to increasing mobility and energy conservation needs, improving bus and coach safety without adding weight is essential. Many crashes with fatal outcomes for vehicle occupants are associated with the rollover of the vehicle, revealing the structural weakness of the steel pillars between windows, which must resist high levels of bending during rollovers. This study aims to reinforce these pillars with expired carbon fiber prepreg from the aircraft industry, improving safety and reducing environmental waste. To manufacture the pillars, shot-blasted hollow S275 steel tubes with a side length of 25 mm and a thickness of 1.5 mm were used. Bidirectional GG600T woven carbon fiber, CF, and aircraft-grade recycled carbon fiber-reinforced plastic, rCFRP, prepreg M21EV/IMA/3 were used as composite reinforcements. The first composite was made from a CF weave using the rigid epoxy resin Sicomin^®^ 8500/Sicomin^®^ SD8601. The rCFRP composite was frayed, and a new composite was made with the same rigid epoxy resin. Both composites were joined to the steel tube using a tough structural adhesive (SikaPower^®^ 1277). A third composite was obtained using the frayed rCFRP and the structural adhesive as a polymer matrix. All composites were treated with an APPT (atmospheric-pressure plasma torch) before being joined to the steel pillar with the structural adhesive. The comparison of the three reinforcements showed that the steel reinforced with the recycled prepreg composite manufactured with the rigid adhesive performed best, with a 50% increase in specific bending strength and only a 32% increase in weight. It also absorbed 71% more energy, which shows that this novel option for upcycling can noticeably increase the crashworthiness of structures.

## 1. Introduction

Road transport is an important link in the value chains of industrialized countries, becoming a fundamental economic and social activity. The increasing mobility of the population, along with the demand for energy efficiency, has led to considerable growth in collective passenger transport by buses and coaches in recent years. The passenger transport vehicle market, particularly for buses and coaches, faces intense pressure due to heightened business competition, stricter safety regulations, and rising user expectations. For coaches, rollover is the most serious type of accident, prompting the development of specific safety regulations [1,2].

**Figure 1:**
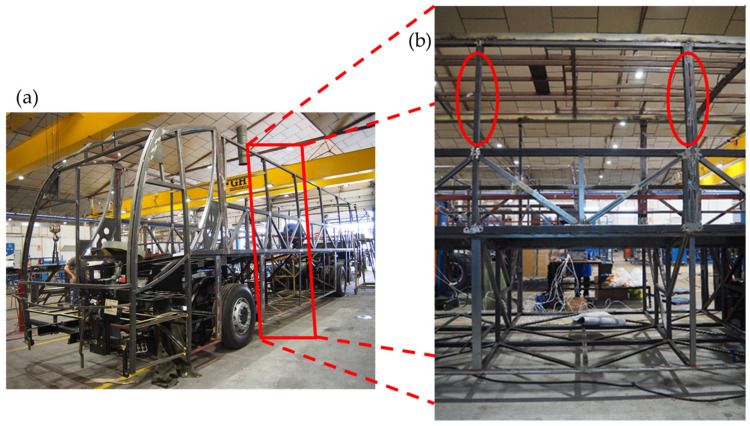
Bus structure: (**a**) steel pillars and (**b**) details corresponding to red area in (**a**), where the pillars are between windows. The red rectangle in figure (**a**) corresponds to the enlarged area in figure (**b**). The red ellipses in figure (**b**) correspond to the weakest area and therefore the most damaged in a rollover. Photos were taken by the authors.

**Figure 2:**
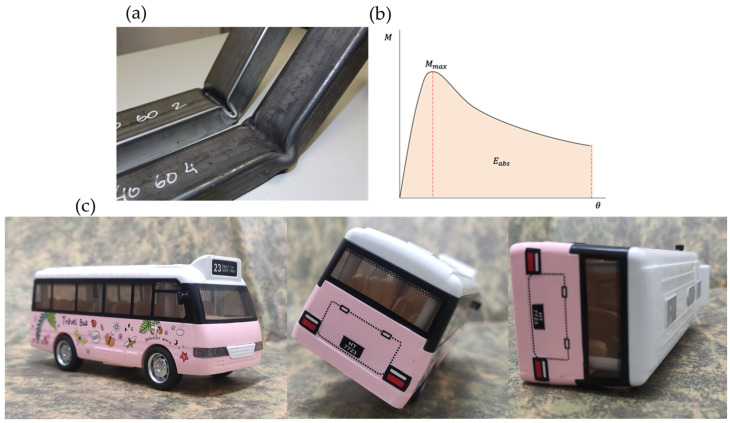
(**a**) Steel tubes after suffering bending collapse. This phenomenon typically occurs at the base of the steel pillars, where most of the plastic strain is concentrated. (**b**) Typical bending moment– angle curve and (**c**) bus overturn sequence. (Own images).

The bus superstructure (Figure 1a) is constructed from thin rectangular steel sections with welded joints. This design offers several well-known advantages: moderate production costs, widely adopted industrial practices, easy assembly, and readily available skilled labor and necessary materials. Geneva Regulation No. 66 [3] states that the vehicle’s superstructure must have sufficient strength to ensure that the survival space is not compromised during or after a rollover test of the complete vehicle. During a rollover, the greatest deformations occur at the junction between the window frames and the profiles beneath them (Figure 1b) [4,5]. This deformation causes the window frames to tilt diagonally, intruding into the passenger’s survival space. This deformation is due to a phenomenon called bending collapse. Thin-walled shapes such as tubes are prone to this phenomenon when subjected to high bending loads [6]. Bending collapse occurs locally in the zones with the largest internal bending moments, such as rigid joints (Figure 2a). In general, their behavior can be evaluated using the characteristic bending moment–angle curve [3] (Figure 2b). Furthermore, this behavior can also be explained by two main characteristics: the bending strength, given by the maximum bending moment in the characteristic curve, and the energy absorbed, given by the area under the load–displacement or bending moment–angle curve [7,8,9] (Figure 2b).

At this point, it is essential to highlight that, although buses can be involved in different types of accidents, rollovers or overturns are the most fatal, resulting in a significant number of fatalities. This risk is heightened by buses’ high center of gravity, considerable height, and weight. Additional factors, such as passenger movement within the bus—especially in school buses with children—further increase the likelihood of overturning. Furthermore, a lack of seatbelt use during a rollover can result in passengers being ejected through the front windshield, leading to fatal injuries. Passengers on the side of the bus that impacts the ground are especially at risk of being crushed between the vehicle’s structure and the pavement [10,11,12].

Increasing the thickness of the steel shapes significantly increases the weight of the structure, leading to higher fuel consumption and manufacturing costs. Therefore, it would be most appropriate to design an optimized reinforcement for these profiles considering the main directions in which they will withstand forces in the event of a rollover. To this end, it is proposed to use a carbon fiber-reinforced polymer (CFRP), using a suitable structural adhesive to join the two dissimilar materials (steel and CFRP) [13,14]. The use of CFRPs has improved fuel efficiency in transportation. This is justified as steel components account for approximately 56% of the weight of a standard passenger vehicle. Replacing them with lightweight materials can lead to weight reductions of between 10 and 70% per part and reduce fuel consumption by between 6 and 42% [15]. Similarly, in the aviation sector, the use of CFRPs has shown significant potential. The projected net reduction in the carbon footprint for 2035 in a Boeing aircraft fleet of 39,620 aircraft is estimated to be approximately 1 × 10^6^ tons of CO_2_ equivalent, associated with a 53.8 wt.% CF percentage [16].

From an economic perspective, the sustainable return on investment in lightweight materials like advanced steel and carbon fiber-reinforced polymers for car bodies depends on manufacturing expenses and fuel/environmental costs. Advanced steel is more favorable for electric vehicles, whereas CFRPs offer a lower return, but this may improve with recycled fibers and clean energy production [17]. However, unlike fuel-based initiatives (e.g., new propulsion systems or biofuels), lightweighting strategies can be seamlessly integrated into existing automotive material supply chains and applied to any vehicle, regardless of its propulsion or fuel system [18].

However, a major drawback is that large amounts of CFRP waste end up in landfills at the end of their life cycle, causing serious environmental and waste management issues. This problem is particularly acute in sectors such as the aerospace, wind energy, and automotive sectors, where CFRP waste is generated in large quantities. From the aeronautical industry alone, 500 kt of CFRP waste is expected to be generated by 2050 [19].

CFRP composites are extensively used across various industries due to their high specific stiffness and strength. Drawing from the aeronautical industry’s experience, other sectors, such as the wind power and automotive industries, have also adopted these materials for both structural components and low-stress parts [20]. This has led to an exponential increase in the use of CFRPs, as well as a corresponding rise in the waste generated by these industries. Driven by environmental regulations, companies are now prioritizing research into recycling and reuse processes for CFRP materials. These processes include various physical, chemical, thermal and mechanical methods [5,21], such as crushing discarded CFRP components [4] to recover short carbon fibers.

As the production of carbon fiber-reinforced composite parts increases in both volume and variety, more versatile and faster manufacturing methods are required. One such approach involves using carbon prepreg waste (CPW) and curing it in an autoclave [22]. CPW consists of a core made from dry carbon fibers sandwiched between upper and lower resin layers [23]. By keeping the resin at low temperatures (−18 °C), it is prevented from flowing between the fibers, significantly slowing down the curing process. Out-of-autoclave CPW has gained acceptance among component manufacturers, as it allows for the production of autoclave-quality parts using vacuum bag-only (VBO) curing methods [24]. This approach helps reduce both facility and production costs.

In the manufacturing of aircraft components, due to the large surface area of the parts and the use of multiple pre-impregnated layers, a significant amount of carbon fiber waste is generated (30 to 50% by weight) [25]. Additionally, due to safety concerns related to the expiration of CPW, entire coils are often discarded [26]. Despite this, mechanical tests have shown that the material’s properties either remain unchanged or show only minor variations (such as slight sensitivity to delamination) even after 9 years of optimal storage [27].

Given the high costs of these materials, there is increasing interest in recycling CPW. CPW chips used in the aeronautical industry, with uncured resin and fibers measuring between 12 and 25 mm in length, have been tested for manufacturing shaped parts using compression molding in a hot plate press [28,29]. Contini et al. [30] explored fiber–matrix separation processes through pyrolysis for expired CPW, though this method results in a reduction in properties compared to virgin fibers. Irez et al. [31] obtained short carbon fibers (1 to 3 mm) through a milling process, which were then used as reinforcement in polypropylene (PP) matrix composites, improving the polymer’s fracture toughness. Similarly, Nosbi et al. [32] used milling to produce short carbon fibers, which were mixed with fiberglass in a PP matrix composite and studied for their water degradation properties.

To maximize the benefits of longer fibers, Butenegro et al. [33] mechanically separated long carbon fibers without removing the epoxy and used them in the manufacturing of composites with a new PA11 matrix. This resulted in significantly improved mechanical properties compared to the matrix alone.

The reuse of carbon fiber waste in the production of new composites not only adds value to these materials but also mitigates potential environmental issues. Considering the solution adopted to enhance safety in buses and coaches, along with the challenges of recycling CFRPs, the objective of this work is to reinforce steel pillars using recycled CFRPs (rCFRPs). For this purpose, expired CFRP prepreg was utilized, without removing the epoxy resin that impregnates it, and mechanically cut (with scissors) to form a new CFRP material that reinforces the structure. While this recycling method has previously been used by the research group in the production of composite materials with a thermoplastic matrix [33,34], the novelty of this work lies in applying it with a thermosetting matrix and bonding it to steel pillars to enhance their bending collapse behavior.

## 2. Materials and Methods

### 2.1. Materials

S275 was the steel used in this work, and it is a low-alloy steel according to UNE-EN 10025 [35]. This steel was supplied by Cerrajería Industrial (Ajalvir, Spain) in a 25 mm × 25 mm × 1.5 mm square tube format. Table 1 shows the maximum percentage levels of certain regulated elements found in European structural steel grade S275. Its minimum yield strength at a nominal thickness of 16 mm is 275 MPa, and it has a tensile strength of 530 MPa for the same thickness [36].

Two types of carbon fiber (CF) were used to prepare CFRPs for reinforcing the steel pillars. The first was bidirectional, balanced GG600T carbon fiber, supplied by Mel Composites in Barcelona, Spain. The mechanical properties of this fiber are a strength (nominal) of 4900 MPa and a modulus (nominal) of 250 GPa, with an areal density of 600 g/m^2^ and a 2/2 twill weave. It was used for comparison with an expired aeronautical prepreg, HexPly M21EV/IMA, provided by Hexcel (Hexcel Composites, Madrid, Spain). This prepreg consists of carbon fibers embedded in a high-performance epoxy resin, originally designed for primary aerospace structures, with a density of 1.28 g/cm³, a glass transition temperature (T_g_) of 180 °C, and a fiber volume of 59.2%. The mechanical properties of the prepreg include a tension strength of 3050 MPa, a longitudinal modulus of 178 GPa, a transverse modulus of 11.8 GPa, a longitudinal Poisson’s ratio of 0.39, and an in-plane shear modulus of 5.2 GPa. Throughout this paper, this prepreg is referred to as rCF.

**Figure 3:**
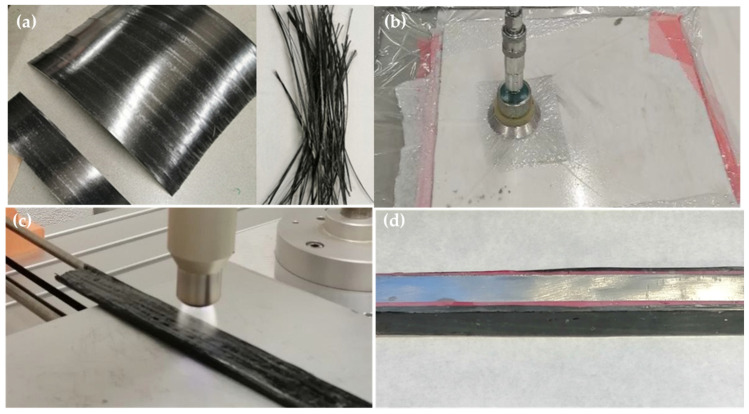
Different steps in the preparation of specimens for bending tests. (**a**) Prepreg and frayed prepreg; (**b**) vacuum bag for curing composites; (**c**) treatment of rigid rCFRP with atmospheric-pressure plasma torch; and (**d**) bending test specimen for steel pillar reinforced with rigid rCFRP.

Two different epoxy systems were used to manufacture the new CFRP and rCFRP. The polymer matrix for the CFRP consisted of Sicomin SR8500 epoxy resin with Sicomin SD8601 hardener (both supplied by Sicomin Epoxy Systems, Châteauneuf-les-Martigues, France) [37]. Sicomin is a versatile epoxy system designed for producing composite parts of all sizes, and it is capable of undergoing continuous operation at temperatures of up to 70 °C. It is known for its rigidity, with a tensile strength of 42 MPa, an elastic modulus of 3390 MPa, and an elongation at break of 1.2% [37]. The CFRP was then bonded to the sides of the steel pillar forms using the two-component adhesive SikaPower 1277 (supplied by Sika SAU, Alcobendas, Spain) [38], which is based on epoxy resin and contains microspheres to ensure uniform thickness. This structural adhesive offers high mechanical strength and is also well suited to impact scenarios. It is classified as tough, with a tensile strength of 30 MPa, an elastic modulus of 2000 MPa and an elongation at break of 4% when tested at 23 °C and 50% RH [38]. Furthermore, two different rCFRPs were manufactured. The first one was manufactured using the Sicomin epoxy system as a matrix and frayed prepreg (Figure 3a); the composite was then joined to the steel shape using the SikaPower 1277 adhesive. The second rCFRP was manufactured using the SikaPower 1277 as a matrix, and the same adhesive was used to join it to the steel pillar.

### 2.2. Manufacturing of Specimens for Mechanical Testing

Square steel tubes were cut into 300 mm samples and degreased using a lanolin-free cloth soaked in isopropyl alcohol, followed by shot blasting with alumina. The samples were then cleaned again with isopropyl alcohol and blown with compressed air.

The CFRP plate was fabricated through hand lay-up of four layers of carbon fiber using the Sicomin epoxy resin. The reinforced tubes that use this composite are labeled as rigid CFRPs in this document.

To prepare the rCFRP, the prepreg was first mechanically reduced (frayed) to a length of 300 ± 10 mm, a width of 40 ± 2 μm, and a thickness of 1.2 ± 0.2 μm (Figure 3a). The size of the rCF rods was previously optimized by Butenegro et al. [33], who found that smaller sizes increase the likelihood of cracks, as they tend to form at the tip of the rCF rod [34]. The rCFRP was then manufactured by hand lay-up, using an equivalent weight of rCF and adhesive to match that used in the production of the CFRP composite. For the rCFRP, two different composite sheets were manufactured, one using Sicomin epoxy resin as a matrix (labeled rigid rCFRP) and the other using the SikaPower 1277 adhesive as a matrix (labeled toughness rCFRP). The sheets from all three types of composites were cured in a vacuum bag for 24 h at room temperature (22 °C) and a pressure of 10^−3^ atm to ensure proper curing (Figure 3b) before proceeding to the next step. These composites were bonded to the steel pillars using SikaPower 1277. A summary of the different combinations is shown in Table 2.

Once cured, the composite sheets were cut into samples measuring 300 mm in length and 20 mm in width and treated with an APPT (atmospheric-pressure plasma torch) to enhance wettability and ensure good adhesion to the steel pillars (Figure 3c).

Each steel pillar was reinforced on the top and bottom faces (Figure 3d) for the 3-point bending test, which are the faces where the force is applied and where deflection occurs during the bending test, corresponding to the position of the reinforcements on the inner and outer faces of the bus pillars. This configuration was optimized by Lavayen-Farfan et al. [39,40] through simulation and bending tests with reinforcements in the flanges and webs.

### 2.3. Atmospheric-Pressure Plasma Torch Treatment

The APPT device employed in this research was developed by Plasmatreat GmbH (Steinhagen, Germany). It operated at a frequency of 17 kHz with a high-voltage discharge of 20 kV and was equipped with a rotating torch, terminating in a nozzle (rotating at 1900 rpm) through which plasma was expelled. The system featured an electronically controlled platform to adjust the speed at which the samples were placed. Plasma was generated inside the rotating nozzle at a working pressure of 2 bars through non-equilibrium discharge and released through a circular aperture onto the samples. The APPT parameters included a torch speed of 3 m/min and a torch-to-sample distance of 6 mm.

As a summary, or based on Figure 3, the following protocol is proposed for the manufacturing of samples for three-point bending tests:

(a) Extract fibers from the prepreg and fray them (Figure 3a).
(b) Manufacture the composite using the extracted fibers with each of the two epoxy resins.
(b’) Manufacture the composite for comparison using bidirectional virgin fabric with the rigid Sicomin adhesive.
(c) Cure the composites in a vacuum bag at room temperature (Figure 3b).
(d) Apply the APPT to the manufactured composites (Figure 3c).
(e) Adhere the reinforcements to the steel pillar, after shot blasting, on the two non-consecutive faces using the tough SikaPower 1277 adhesive.

### 2.4. Bending Test Specimen

The three-point bending test was selected to evaluate the bending strength and energy absorption capabilities of each type of reinforcement. A universal testing machine supplied by Microtest (Madrid, Spain) with a 200 kN load capacity was employed. The load was applied at the center using an applicator moving at a rate of 100 mm/min. The support cylinders remained fixed throughout the experiment.

The distance between supports, L, must be sufficiently large to ensure bending collapse without indentation; specifically, the span (L) to height (b) ratio must be larger than 8, i.e., L/b > 8 [41]. Given that the test specimens had sides of 25 mm, a support distance of 250 mm was appropriate. Also, to obtain a baseline result, a tube with no reinforcement was tested. It is worth noting that should indentation occur, it would render the results useless since the phenomenon that needs to be characterized is bending collapse.

The testing machine records data on the applied force and the generated displacement, which can be used to plot a force (F) versus displacement (δ) curve. The original curves were used to calculate the maximum bending resistance (σ) and elongation (ϵ), according to Equations (1) and (2). For the square tube, the width and height are the same (b), but the height must be added to twice the thickness of the composite (a). These parameters allow for determining whether the passenger compartment of the coach or bus could be compromised in the event of a collision or rollover.

(1) $$\sigma =\frac{3\times F\times L}{2\times {b\times (b+a)}^{2}}$$

(2) $$\epsilon =\frac{6\times \delta \times (b+a)}{L^{2}}$$

Then, the measured force, F, was converted into a bending moment (M), and the displacement, δ, was converted into a rotation or bending angle (θ). This conversion was performed using standard equations from technical mechanics for a simply supported beam under a centrally applied transversal load, as shown in Equations (3) and (4). This conversion is needed to eliminate the influence of the span (L) on the results and to obtain a metric that can be compared to other works in the literature. Furthermore, the collapse in the middle of the specimen occurs due to bending collapse, which, in turn, is provoked by bending moments.

(3) $$M=\frac{F\times L}{4}$$

(4) $$\theta =2\tan^{-1}\left(\frac{2\delta }{L}\right)$$

**Figure 4:**
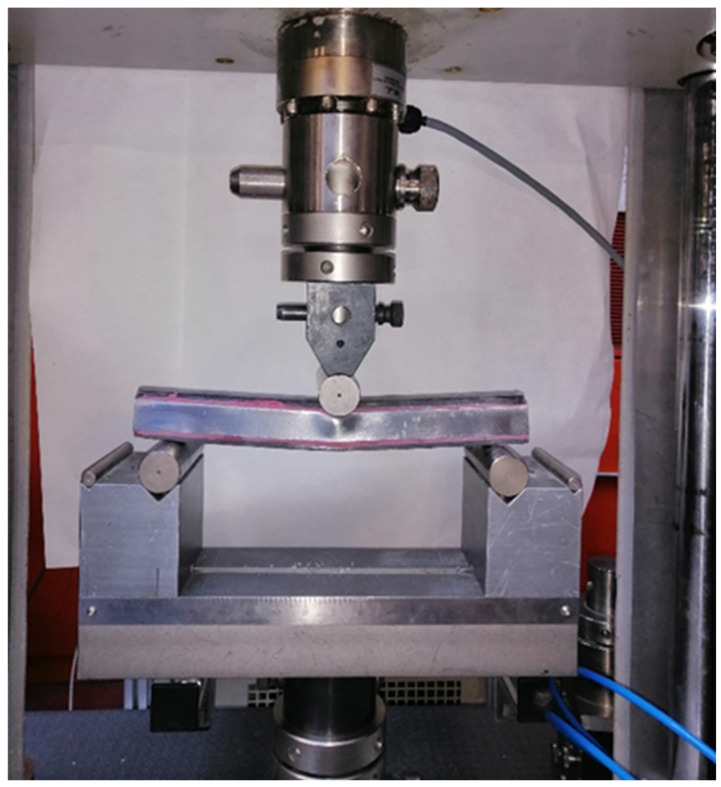
Bending test machine: steel pillar reinforcement with rigid rCFRP.

Three tests for each structure were carried out and compared to the unreinforced steel pillar. Figure 4 shows the three-point bending test.

## 3. Results

**Figure 5:**
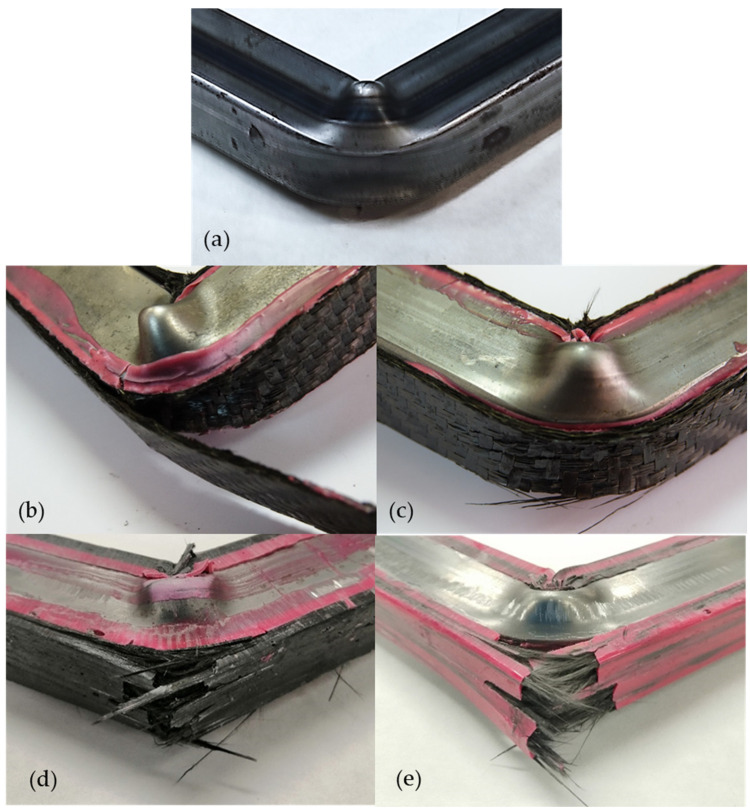
Bending specimens tested for up to 60 mm of rotation: (**a**) steel pillar; (**b**) delamination of rigid CFRP; (**c**) fracturing of rigid CFRP composite; (**d**) fracturing of rigid rCFRP composite; and (**e**) fracturing of toughened rCFRP composite.

Figure 5 shows the rotation of the different bending samples, with the test always stopping at 100 mm of displacement. The bending could not be extended to greater displacements as the sample would become detached from the supports. At this displacement, the steel pillar has already collapsed without fracturing. The load is applied through the support points of the testing machine, which create friction with the composite reinforcement material. The tested steel pillar (Figure 5a) serves as a comparison with the reinforced pillars. Delamination was observed only in some cases with the rigid CFRP composite (Figure 5b), while fracturing of the laminate occurred in other samples (Figure 5c). In the case of the rCFRP composite manufactured with a rigid (Figure 5d) or tough (Figure 5e) matrix, fracturing of the laminate was consistently observed.

**Figure 6:**
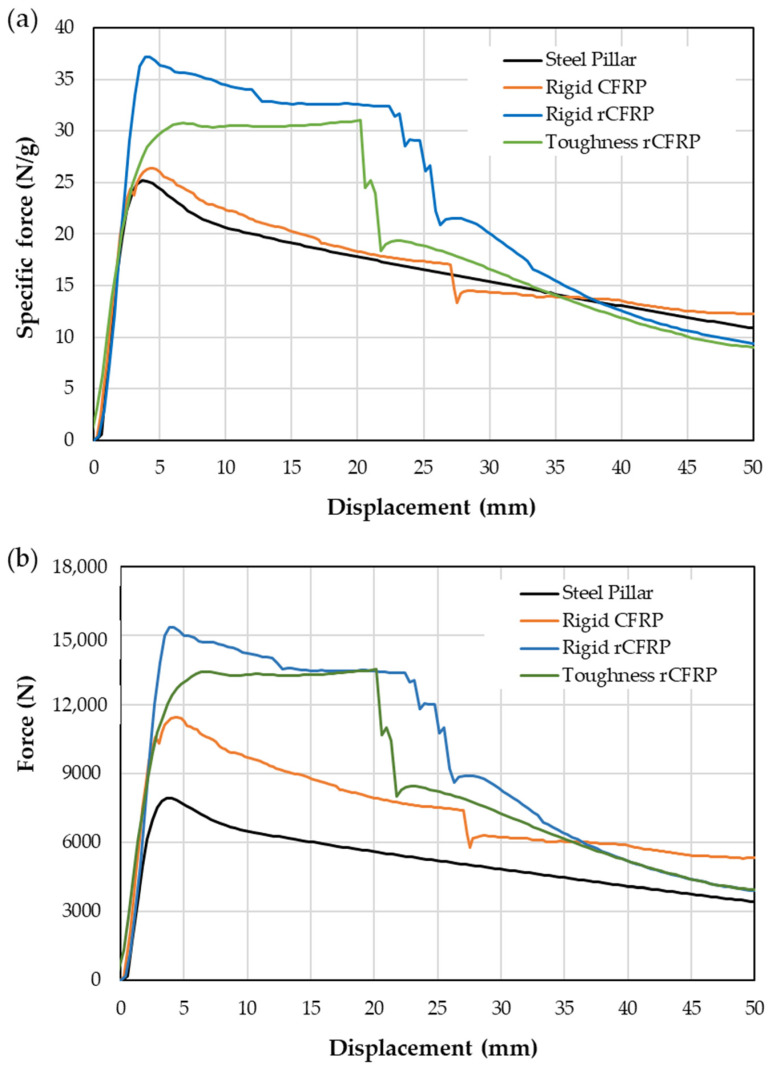
Three-point bending data: (**a**) specific force–displacement and (**b**) force–displacement for steel pillar and reinforced steel pillar. All the tests are stopped at 100 mm of displacement, although the graphics only show the initial part of the curves up to 50 mm to better observe the differences between the different reinforcements.

The results for bending strength and deformation, calculated using Equations (1) and (2), did not yield data that could differentiate between the different configurations relative to the steel. The force–displacement (F-δ) graph provides useful information on the behavior of the composite. However, to include the influence of the weight of the composite, specific force (force per unit mass)–displacement plots are also given (Figure 6a). Furthermore, the energy absorption rates of the specimens can be calculated from the force–displacement curve (Figure 6b), both at maximum force and at a given displacement.

The three reinforced steel pillars exhibited steps or “jumps” in the specific force–displacement curves, corresponding to the fracturing of the reinforcement (Figure 6a). In the case of the steel pillar–rigid rCFRP, this step corresponds to a drop of 16 N/g in a displacement range of 21 mm, with smaller steps clearly observed between 23 mm and 26 mm of displacement, corresponding to the fracturing of each layer. For the steel pillar–toughness rCFRP, the specimens tend to hold a specific force of around 30 N/g (a desirable trait) but have a rapid drop off at around 21 mm. On the other hand, the curve for the steel pillar–rigid CFRP is more similar to that of the unreinforced steel pillar, showing a very small step (−5 N/g) at a displacement of 27 mm. The steel pillar–rigid rCFRP showed a higher specific force, together with the steel pillar–toughness rCFRP, than the steel pillar without reinforcement and steel pillar–rigid CFRP (Figure 6a). Furthermore, the steel pillar–rigid CFRP presented a greater force than the steel pillar with no reinforcement (Figure 5b), although its weight was similar to the steel pillar–toughness rCFRP: 433 g and 436 g, respectively. On the other hand, steel pillar–rigid CFRP maintained a high value of specific force for up to 26 mm. This means that greater strength and crashworthiness can be obtained without a significant increase in weight.

If the weight of the reinforcement is not taken into account (Figure 6b), additional differences between the steel pillar and the steel pillar–rigid CFRP can be observed. This is due to the weight difference, from 314 g for the unreinforced pillar to 433 g for the CFRP-reinforced pillar. However, the bending strength of the pillar is clearly greater when the rCFRP is applied (Figure 6b). From the force–displacement curves (Figure 6b), the energy absorbed at the maximum force or at a given displacement is calculated, which can also be used to compare different materials.

**Figure 7:**
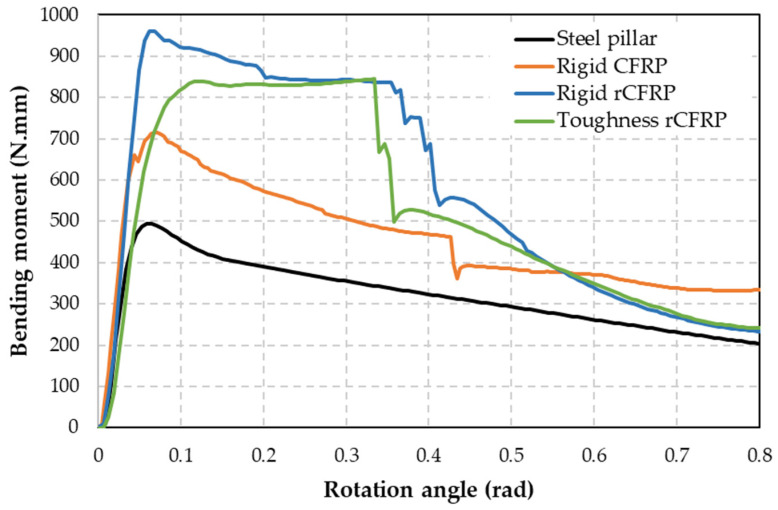
Bending moment–rotation angle for steel pillar and reinforced steel pillar.

Most studies on hollow tubes or beams related to three-point bending tests use the bending moment–rotation or bending angle curves (Figure 7) for comparison between different specimen dimensions, since both are independent of the tube dimensions. These parameters have also been used by multiple researchers as metrics for strength and energy absorption [6,39,40,41,42,43]. This approach also helps in simulating this type of test and comparing the results with most of the studies in the bibliography, and most importantly, this is a key factor for the requirements of regulations and standards such as Regulation 66 [3].

Table 3 lists all the parameters measured in these three-point bending tests. The only parameters that decrease in relation to the steel pillars are the maximum bending strength and the bending modulus. When comparing the composite using virgin CF for reinforcement with those using rCF, the rCF performs better. For example, a 32% increase in the weight of the steel pillar with the rigid rCFRP compared to the other steel pillar resulted in a 50% increase in force, a 10% increase in displacement, a 117% increase in the energy absorbed at maximum strength, a 68% increase in the bending angle, a 4% increase in the maximum strength, a 53% increase in strain, and a 71% increase in the energy absorbed at 10 cm of bending, while the bending modulus decreased by 32%. The differences between virgin CF and rCF can only be attributed to the presence of epoxy resin in rCF, as it comes from expired prepreg.

## 4. Discussion

Three different types of reinforcements for structural steel tubes were tested and compared. The reinforcements have the novelty of being manufactured using a recycled CFRP, or rCFRP, with two different manufacturing techniques. In general, the rCFRP offers better improvements and better crashworthiness than reinforcements with virgin CF and unreinforced tubes.

One key reason for this difference and the superior performance of both rCFRPs can be attributed to the fiber orientation within each composite. In the case of the virgin CF, the bidirectional weave means that half of the fibers are oriented perpendicular to the normal stresses that occur due to bending loads. This means that this composite is not being fully used. On the other hand, both rCFRPs are manufactured using frayed fibers from expired prepregs and reoriented in one direction. As this direction is aligned with the normal stress due to the bending loads, all or most of the fibers are fully loaded, which explains the greater strength and energy absorption. On the other hand, the frayed composite may have allowed for better wetting, adhesion, and, ultimately, load transfer between the matrix and fibers. 

Furthermore, since the rigid CFRP (using virgin CF) was built using hand lay-up, the loads between layers were transferred by the matrix in the form of shear stresses [26], which is evidenced by the specimens that failed due to delamination (Figure 5b). Shear stress is produced when the layers try to slip relative to one another, and the matrix holds them in place. On the contrary, the rCFRP is built as “one layer”; thus, there is no slip. This is further evidenced by the failure modes observed, as both the epoxy resin and epoxy adhesive were able to successfully transfer the load to the frayed composite in order for it to withstand the load. In all cases, fracturing on the tension zone led the composite to fracture (Figure 5d,e). Even when both rCFRPs outperform the virgin CFRP, the rigid rCFRP (made with epoxy resin as the matrix) has a slight advantage over the toughness CFRP (made with adhesive as the matrix). This can be explained by the microspheres in the adhesive, which are used to maintain a uniform thickness and take up space between the matrix and the fibers, thus slightly reducing the wettability. In this sense, these types of adhesives should be avoided if used as a matrix for a fiber composite.

Since a specific analysis of reinforcements with these characteristics in bus structures or other structures has not been carried out in any previous study found in the literature, it makes it very difficult to propose a direct comparison, yet there are some studies that can be mentioned. Thus, Haedir et al. [42] reported an increase in the bending resistance in structures when using carbon fiber-reinforced polymer sheets to reinforce circular hollow steel sections. In addition, CFRP sheets are also used to reinforce short columns of circular steel tubes due to their ease of handling and light weight. High-strength CFRP sheets can provide a degree of restraint to delay the buckling of a thin steel wall, improving the axial section capacity of the steel tube [43].

The use of fiber-reinforced polymers in columns is rising due to their roles in confinement, reinforcement, and structure. Fiber orientation is key: transverse for confinement and axial for compression. Multidirectional fibers enhance stiffness in both directions [44]. Thus, carbon fiber (CF) was explored as a reinforcement material in CF/PEEK (polyether–ether–ketone) tubes, using induction heating and rotary stretch bending. This technique ensures proper formation despite the high melting point of PEEK using a spring steel mandrel. Changes in fiber angle were analyzed, confirming the effectiveness of CF in maintaining structural integrity [45]. Models are also available to optimize laminate design, taking fiber angle changes into account [46].

These findings suggest that reinforcing metallic and polymeric structures with CF consistently enhances performance, provided the CF arrangement in the composite is carefully considered. In this study, the laminate placement on the steel pillar was already optimized. Therefore, the evaluation focused solely on the use of expired prepreg and adhesive, maintaining the same design.

Lastly, the results of this study demonstrate the enormous potential of recycled composites, particularly rCFRPs, in fostering a more sustainable and circular economy. The reuse of expired aerospace prepregs can help mitigate challenges in other industries where components are subjected to high loads while also reducing carbon fiber waste. There is a general consensus that the high costs of fiber composites may limit their wider use as structural components. By upcycling CFRP waste, the costs associated with both materials and manufacturing can be reduced. This approach not only aligns with the principles of a circular economy but also offers an interesting avenue for future research, with the potential to enhance safety in buses and other applications.

## 5. Conclusions

This study compares three types of reinforcements for structural steel pillars, emphasizing the superior performance of recycled carbon fiber-reinforced polymer (rCFRP) reinforcements in terms of strength and energy absorption.

Rigid rCFRP reinforcements demonstrate a 9% increase in maximum strength and a 45% improvement in energy absorption compared to virgin carbon fiber composite (CFRP) reinforcements. When compared to unreinforced steel pillars, they show a 4% increase in strength and absorb 117% more energy at maximum strength. These enhancements are attributed to optimized fiber orientation, which improves the load-bearing capacity.

Tough rCFRP reinforcements excel in energy absorption, achieving 264% more energy absorption than unreinforced steel pillars and 192% more than steel pillars with CFRP reinforcements. However, their maximum strength decreases by 19% relative to unreinforced steel pillars and by 14% compared to CFRP-reinforced pillars.

The flexural modulus of reinforced steel pillars is consistently lower, indicating reduced stiffness, while the reinforcement adds 32% to 39% more weight. Despite this, the single-layer rCFRP construction enhances load transfer between fibers and the matrix, minimizing delamination issues.

These findings underscore the potential of recycled composites, particularly rCFRPs, to foster sustainability, lower material costs, and support a circular economy by repurposing aerospace-grade materials.

## Figures and Tables

**Table 1:** The chemical composition of S275 structural steel.

Steel	C%	Mn%	P%	S%	Si%
S275	0.225 max	1.60 max	0.04 max	0.05 max	0.05 max

**Table 2:** Different combinations of manufacturing composite materials.

Resin (Matrix)	CF	rCF	Bonding Sika Power 1277
Sicomin SR8500/SD8601	Rigid CFRP	-	Steel pillar–rigid CFRP
Sicomin SR8500/SD8601	-	Rigid rCFRP	Steel pillar–rigid rCFRP
SikaPower 1277	-	Toughness rCFRP	Steel pillar–toughness rCFRP

**Table 3:** Parameters calculated and obtained from the three-point bending tests.

Parameters	Steel Pillar	Steel Pillar– Rigid CFRP		Steel Pillar– Rigid rCFRP		Steel Pillar– Toughness rCFRP	
	Value	Value	% *	Value	% *	Value	% *
Force max. (N)	7904	11,560	+46	15,595	+97	14,391	+82
Weight (g)	314	433	+38	413	+32	436	+39
Force max./weight (N/g)	25	27	+6	38	+50	33	+31
Displacement (mm)	3.66	4.33	+18	4.03	+10	7.32	+100
Energy at the max. (J)	15	25	+72	31	+117	53	+264
Angle at the max. (rad)	0.059	0.069	+18	0.099	+68	0.123	+110
Strength max. (MPa)	189.69	180.42	−5	197.54	+4	154.32	−19
Strain at the max (%)	0.88	1.29	+47	1.34	+53	2.63	+200
Modulus (GPa)	21.63	14.37	−34	14.76	−32	5.93	−73
Energy at 10 cm (J)	61	90	+48	104	+71	107	+76

* Variation with respect to the steel pillar.

## Data Availability

The raw data supporting the conclusions of this article will be made available by the authors upon request.

## References

[1] Buses and Coaches—Road Safety—European Commission. https://road-safety.transport.ec.europa.eu/.

[2] Karliński J., Ptak M., Działak P., Rusiński E. (2014). Strength Analysis of Bus Superstructure According to Regulation No. 66 of UN/ECE. Arch. Civ. Mech. Eng..

[3] European Parliament Council of the European Union (2010). Regulation No 66 of the Economic Commission for Europe of the United Nations (UN/ECE)—Uniform Provisions Concerning the Approval of Large Passenger Vehicles with Regard to the Strength of Their Superstructure. Off. J. Eur. Union.

[4] Aldosari S.M., AlOtaibi B.M., Alblalaihid K.S., Aldoihi S.A., AlOgab K.A., Alsaleh S.S., Alshamary D.O., Alanazi T.H., Aldrees S.D., Alshammari B.A. (2024). Mechanical Recycling of Carbon Fiber-Reinforced Polymer in a Circular Economy. Polymers.

[5] Butenegro J.A., Bahrami M., Abenojar J., Martínez M.Á. (2021). Recent Progress in Carbon Fiber Reinforced Polymers Recycling: A Review of Recycling Methods and Reuse of Carbon Fibers. Materials.

[6] Lavayen-Farfan D., Boada M.J.L., Rodriguez-Hernandez J.A. (2021). Bending Collapse Analysis for Thin and Medium-Thin-Walled Square and Rectangular Hollow Shapes. Thin-Walled Struct..

[7] David-West O.S., Alexander N.V., Nash D.H., Banks W.M. (2008). Energy Absorption and Bending Stiffness in CFRP Laminates: The Effect of 45° Plies. Thin-Walled Struct..

[8] Wu Z., Shen Y., Pan Z., Hu X. (2019). Three-Point Bending Behavior and Energy Absorption Capacity of Composite Tube Reinforced by Gradient Braided Structure in Radial Direction. Fibers Polym..

[9] An D., Liu T., Cui H., Chen Z., Xu H., Song Y. (2022). Study of the Factors Influencing Load Displacement Curve of Energy Absorbing Device by Area Division Simulation. Sci. Rep..

[10] NHTSA Traffic Safety Facts 2021 Data. https://crashstats.nhtsa.dot.gov/Api/Public/ViewPublication/813473.

[11] Nicolás Fraile C. On the Bus, Also Buckled Upo. https://www.google.com/url?sa=t&source=web&rct=j&opi=89978449&url=https://revista.dgt.es/Galerias/noticia/nacional/2014/11NOVIEMBRE/num199-2009-autocares.pdf&ved=2ahUKEwjXub7YrJCKAxXNAfsDHSuCPUwQFnoECBcQAQ&usg=AOvVaw0LmXXEn5zXxJ6jVwWAs3xI.

[12] Wekezer J.W., Cichocki K. (2007). Structural Response of Paratransit Buses in Rollover Accidents. Int. J. Crashworthiness.

[13] Kah P., Suoranta R., Martikainen J., Magnus C. (2014). Techniques for Joining Dissimilar Materials: Metals and Polymers. Rev. Adv. Mater. Sci..

[14] Galvez P., Quesada A., Martinez M.A., Abenojar J., Boada M.J.L., Diaz V. (2017). Study of the Behaviour of Adhesive Joints of Steel with CFRP for Its Application in Bus Structures. Compos. Part B Eng..

[15] Pervaiz M., Panthapulakkal S., KC B., Sain M., Tjong J. (2016). Emerging Trends in Automotive Lightweighting through Novel Composite Materials. Mater. Sci. Appl..

[16] Khalil Y.F. (2017). Eco-Efficient Lightweight Carbon-Fiber Reinforced Polymer for Environmentally Greener Commercial Aviation Industry. Sustain. Prod. Consum..

[17] Shanmugam K., Gadhamshetty V., Yadav P., Athanassiadis D., Tysklind M., Upadhyayula V.K.K. (2019). Advanced High-Strength Steel and Carbon Fiber Reinforced Polymer Composite Body in White for Passenger Cars: Environmental Performance and Sustainable Return on Investment under Different Propulsion Modes. ACS Sustain. Chem. Eng..

[18] Das S., Graziano D., Upadhyayula V.K.K., Masanet E., Riddle M., Cresko J. (2016). Vehicle Lightweighting Energy Use Impacts in U.S. Light-Duty Vehicle Fleet. Sustain. Mater. Technol..

[19] Meng F., Cui Y., Pickering S., McKechnie J. (2020). From Aviation to Aviation: Environmental and Financial Viability of Closed-Loop Recycling of Carbon Fibre Composite. Compos. Part B Eng..

[20] Rijo B., Dias A.P.S., Carvalho J.P.S. (2023). Recovery of Carbon Fibers from Aviation Epoxy Composites by Acid Solvolysis. Sustain. Mater. Technol..

[21] Ateeq M. (2023). A State of Art Review on Recycling and Remanufacturing of the Carbon Fiber from Carbon Fiber Polymer Composite. Compos. Part C Open Access.

[22] Das T.K., Ghosh P., Das N.C. (2019). Preparation, Development, Outcomes, and Application Versatility of Carbon Fiber-Based Polymer Composites: A Review. Adv. Compos. Hybrid Mater..

[23] Grunenfelder L.K., Centea T., Hubert P., Nutt S.R. (2013). Effect of Room-Temperature out-Time on Tow Impregnation in an out-of-Autoclave Prepreg. Compos. Part A Appl. Sci. Manuf..

[24] Centea T., Grunenfelder L.K., Nutt S.R. (2015). A Review of Out-of-Autoclave Prepregs—Material Properties, Process Phenomena, and Manufacturing Considerations. Compos. Part A Appl. Sci. Manuf..

[25] Bianchi I., Forcellese A., Marconi M., Simoncini M., Vita A., Castorani V. (2021). Environmental Impact Assessment of Zero Waste Approach for Carbon Fiber Prepreg Scraps. Sustain. Mater. Technol..

[26] Nilakantan G., Nutt S. (2015). Reuse and Upcycling of Aerospace Prepreg Scrap and Waste. Reinf. Plast..

[27] Amare C., Mantaux O., Gillet A., Pedros M. (2023). Simplification of Requalification Procedure of Outdated Carbon/Epoxy Prepregs and Scenarios of Reuse. J. Phys. Conf. Ser..

[28] Smith A.W., Hubert P. (2023). A Novel Recycling Framework for Transforming Uncured Aerospace Prepreg Offcuts into a Strand-Based Compression Moulding Compound with Adjustable Flow-Compaction and Curing Behaviours. Compos. Part A Appl. Sci. Manuf..

[29] Qian C., Yuan H., Begum H., Hill T., Groves R., Joesbury A., Harper L. (2024). Experimental Characterisation for Compression Moulding of Hybrid Architecture Composites Using Reclaimed Prepreg Manufacturing Waste. Mater. Res. Proc..

[30] Sales-Contini R.C.M., Costa H.M.S., Bernardi H.H., Menezes W.M.M., Silva F.J.G. (2024). Mechanical Strength and Surface Analysis of a Composite Made from Recycled Carbon Fibre Obtained via the Pyrolysis Process for Reuse in the Manufacture of New Composites. Materials.

[31] Irez A.B., Yakar H. (2024). Transformation of Waste Carbon Fiber Prepreg into Sustainable Composites: Application in the Automotive Industry Components. Polym. Compos..

[32] Nosbi N., Fadli H., Marzuki A., Zakaria M.R., Fahmin W., Wan F., Javed F., Ibrar M. (2020). Prepreg Waste-Polymer Hybrid Composites Degradation in Water Condition. Processes.

[33] Butenegro J.A., Bahrami M., Swolfs Y., Ivens J., Martínez M.Á., Abenojar J. (2023). Novel Sustainable Composites Incorporating a Biobased Thermoplastic Matrix and Recycled Aerospace Prepreg Waste: Development and Characterization. Polymers.

[34] Butenegro J.A., Bahrami M., Martínez M.Á., Abenojar J. (2023). Reuse of Carbon Fibers and a Mechanically Recycled CFRP as Rod-like Fillers for New Composites: Optimization and Process Development. Processes.

[35] (2020). Hot Rolled Products of Structural Steels—Part 2: Technical Delivery Conditions for Non-Alloy Structural Steels 2020.

[36] Gilbert N. Structural Steel—S235, S275, S355 Chemical Composition, Mechanical Properties and Common Applications. https://www.azom.com/article.aspx?ArticleID=6022.

[37] (2014). Techinal Datasheet Epoxy Resin Sicomin SR 8500 and SD 8601.

[38] (2022). Technical Datasheet SikaPower^®^-1277.

[39] Lavayen-Farfan D., Butenegro-Garcia J.A., Boada M.J.L., Martinez-Casanova M.A., Rodriguez-Hernandez J.A. (2022). Theoretical and Experimental Study of the Bending Collapse of Partially Reinforced CFRP–Steel Square Tubes. Thin-Walled Struct..

[40] Lavayen-Farfán D., Butenegro-García J.A., López-Boada M.J., Martínez Casanova M.Á., Rodriguez-Hernandez J.A., Vizán-Idoipe A., Gracía-Prada J.C. (2022). On the Use of Carbon Fiber Composites for the Enhancement of the Rollover Resistance of Steel Buses. XV Ibero-American Congress of Mechanical Engineering—Advances in Mechanical Engineering.

[41] Huang Z., Zhang X. (2018). Three-Point Bending Collapse of Thin-Walled Rectangular Beams. Int. J. Mech. Sci..

[42] Haedir J., Zhao X.L., Bambach M.R., Grzebieta R.H. (2010). Analysis of CFRP Externally-Reinforced Steel CHS Tubular Beams. Compos. Struct..

[43] Haedir J., Zhao X.L. (2011). Design of Short CFRP-Reinforced Steel Tubular Columns. J. Constr. Steel Res..

[44] Al-saadi A.U., Aravinthan T., Lokuge W. (2018). Structural Applications of Fibre Reinforced Polymer (FRP) Composite Tubes: A Review of Columns Members. Compos. Struct..

[45] Li M., Stokes-Griffin C., Compston P. (2024). Post-Forming of Carbon Fibre-Reinforced PEEK Thermoplastic Tubular Structures. J. Compos. Sci..

[46] Li M., Stokes-Griffin C., Sommacal S., Compston P. (2022). Fibre Angle Prediction for Post-Forming of Carbon Fibre Reinforced Composite Tubular Structures. Compos. Part A Appl. Sci. Manuf..

